# Reassessment of CXCR4 Chemokine Receptor Expression in Human Normal and Neoplastic Tissues Using the Novel Rabbit Monoclonal Antibody UMB-2

**DOI:** 10.1371/journal.pone.0004069

**Published:** 2008-12-31

**Authors:** Thomas Fischer, Falko Nagel, Stefan Jacobs, Ralf Stumm, Stefan Schulz

**Affiliations:** 1 Department of Pharmacology, Julius-Maximilians-University, Würzburg, Germany; 2 Department of Pharmacology and Toxicology, Friedrich-Schiller-University, Jena, Germany; 3 Department of Pharmacology and Toxicology, Otto-von-Guericke-University, Magdeburg, Germany; Washington University School of Medicine, United States of America

## Abstract

**Background:**

The CXCR4 chemokine receptor regulates migration and homing of cancer cells to specific metastatic sites. Determination of the CXCR4 receptor status will provide predictive information for disease prognosis and possible therapeutic intervention. However, previous attempts to localize CXCR4 using poorly characterized mouse monoclonal or rabbit polyclonal antibodies have produced predominant nuclear and occasional cytoplasmic staining but did not result in the identification of bona fide cell surface receptors.

**Methodology/Principal Findings:**

In the present study, we extensively characterized the novel rabbit monoclonal anti-CXCR4 antibody (clone UMB-2) using transfected cells and tissues from CXCR4-deficient mice. Specificity of UMB-2 was demonstrated by cell surface staining of CXCR4-transfected cells; translocation of CXCR4 immunostaining after agonist exposure; detection of a broad band migrating at *M*_r_ 38,000–43,000 in Western blots of homogenates from CXCR4-expressing cells; selective detection of the receptor in tissues from CXCR4+/+ but not from CXCR4−/− mice; and abolition of tissue immunostaining by preadsorption of UMB-2 with its immunizing peptide. In formalin-fixed, paraffin-embedded human tumor tissues, UMB-2 yielded highly effective plasma membrane staining of a subpopulation of tumor cells, which were often heterogeneously distributed throughout the tumor. A comparative analysis of the mouse monoclonal antibody 12G5 and other frequently used commercially available antibodies revealed that none of these was able to detect CXCR4 under otherwise identical conditions.

**Conclusions/Significance:**

Thus, the rabbit monoclonal antibody UMB-2 may prove of great value in the assessment of the CXCR4 receptor status in a variety of human tumors during routine histopathological examination.

## Introduction

The CXCR4 chemokine receptor is a plasma membrane receptor that regulates an array of trafficking events during organogenesis, hematopoesis and inflammation [1]. The specific ligand to CXCR4, stromal cell-derived factor 1 (SDF-1, CXCL12), is expressed at high levels in lymph nodes, lungs, bone marrow and liver. Recent studies indicate that CXCR4 is one of the critical factors for metastasis homing on specific organ sites in that SDF-1 released in target organs may attract nearby or distant CXCR4-expressing cancer cells [2]. Inhibition of the CXCR4-SDF-1-axis by specific CXCR4 antagonists or RNA interference has been shown to block *in vitro* invasion and *in vivo* metastasis of cancer cells in animal models [3], [4], [5]. In fact, expression of CXCR4 in a number of human malignancies has been shown to increase the risk for recurrence and poor survival [6], [7], [8], [9]. Thus, an accurate assessment of the CXCR4 status of a given tumor specimen would provide valuable predictive information for disease prognosis and possible therapeutic intervention.

Consequently, much attention has been directed towards the detection and localization of CXCR4 receptors in human primary tumors. Earlier studies have assessed CXCR4 expression using reverse transcription-polymerase chain reaction (RT-PCR) [2], [6]. However, the diagnostic value of this method is limited. RT-PCR is based on total RNA isolation from a fresh tumor sample and would therefore not only detect CXCR4 receptor transcripts originating from tumor cells but also from lymphocytes, endothelial cells or other non-malignant cells. Other studies have utilized the mouse monoclonal antibodies {12G5} and {44716} for immunohistochemical detection of CXCR4 in human formalin-fixed, paraffin-embedded tumors [7], [8], [9], [10], [11], [12], [13], [14], [15], [16], [17], [18], [19], [20]. These antibodies have been generated by immunizing mice with live CXCR4-expressing cells and presumably bind to an extracellular domain of the receptor [21], [22]. Unfortunately, specific epitope information is not available for {12G5} and {44716}, which eliminates the possibility to perform adsorption controls during immunohistochemical staining. Although flow cytometric analysis suggests that {12G5} and {44716} can bind to CXCR4 on native cells, these antibodies have not been adequately characterized using fixed cells or tissues [21], [22]. In fact, virtually all-previous studies using these and other commercially available antibodies have reported predominant staining of cell nuclei with occasional cytoplasmic staining in human fixed-embedded tissues [7], [8], [9], [10], [11], [12], [13], [14], [15], [16], [17], [18], [19], [20], [23]. Given our understanding of CXCR4 signaling, an entirely nuclear localization would not be compatible with a function of this receptor in cancer cell migration and homing [1].

In the present study, we have extensively characterized the new rabbit monoclonal anti-CXCR4 antibody UMB-2, which is directed against the carboxyl-terminal tail of the receptor. We demonstrate that UMB-2 selectively detects its cognate receptor in fixed cells and tissues. In contrast to currently available monoclonal and polyclonal antibodies, UMB-2 efficiently detects bona fide CXCR4 plasma membrane receptors. Thus, the development of UMB-2 will now allow the establishment of guidelines for routine performance of CXCR4 immunohistochemistry in human tumors.

## Materials and Methods

### Antibodies

Rabbit polyclonal anti-CXCR4 antibodies {2144} and {1181} were generated against the following sequence KGKRGGHSSVSTESESSSFHSS, which corresponds to residues 338–359 of the human CXCR4 receptor. This sequence is identical in mouse, rat and human CXCR4 receptors. Anti-CXCR4 antibodies {2144} and {1181} have been extensively characterized previously in mouse and rat tissues [24], [25], [26], [27], [28]. Rabbit monoclonal anti-CXCR4 antibody clone UMB-2 was generated against the identical sequence and obtained from Epitomics (Burlingame, CA). Rabbit polyclonal anti-CCR7 {1188} was generated against the following sequence CRHIRRSSMSVEAETTTTFSP, which corresponds to residues 358-378 of the human CCR7 receptor. Anti-CCR7 antibody was then affinity purified against its immunizing peptide. The mouse monoclonal anti-CXCR4 antibodies {12G5} and {44716} were obtained from R&D Systems (Minneapolis, MN). The goat polyclonal anti-CXCR4 antibody {6190} was obtained from Santa Cruz Biotechnology (Santa Cruz, CA).

### Tumor Samples

The following tumors were investigated: breast carcinoma (n = 36); ovarian carcinoma (n = 22); cervical carcinoma (n = 16); endometrial carcinoma (n = 4); gastric cancer (n = 13), colorectal adenocarcinoma (n = 23); pancreatic adenocarcinoma (n = 29); prostate cancer (n = 24); carcinoid (n = 18), growth hormone-secreting pituitary adenoma (n = 8); pheochromocytoma (n = 20); glioblastoma multiforme (n = 18); astrocytoma grade II (n = 8); astrocytoma grade III (n = 8). All tissue specimens had been fixed in formalin and were then embedded in paraffin.

### Immunocytochemistry

Plasmids encoding the human CXCR4 and human CCR7 receptors were obtained from UMR cDNA Resource Center (Rolla, MO). Human embryonic kidney 293 (HEK-293) cells were stably transfected with either CXCR4 or CCR7. Cells were grown on coverslips overnight and either not exposed or exposed to 100 ng/ml SDF-1 or 100 ng/ml MIP-3 (R&D Systems). Cells were then fixed and incubated with anti-CCR7 {1188, 1 µg/ml}, anti-CXCR4 {UMB-2, hybridoma supernatant at a dilution of 1∶100}, anti-CXCR4 {6190, 1–10 µg/ml}, anti-CXCR4 {44716, 5–15 µg/ml} or anti-CXCR4 {12G5, 1–25 µg/ml} antibodies followed by the appropriate cyanine 3.18-conjugated secondary antibodies (Amersham, Braunschweig, Germany). Specimens were mounted and examined using a Leica TCS-NT laser scanning confocal microscope as described [29].

### Western blot analysis

HEK-293 cells stably transfected with CCR7 or CXCR4 as well as brains from CXCR4-deficient mice and their wild-type littermates were lysed in detergent buffer (20 mM Hepes, pH 7.4, 150 mM NaCl, 5 mM EDTA, 1% Triton X-100, 10% glycerol, 0.1% SDS, 0.2 mM phenylmethylsulfonylfluoride, 10 mg/ml leupeptin, 1 mg/ml pepstatin A, 1 mg/ml aprotinin and 10 mg/ml bacitracin) and glycoproteins were enriched using wheat germ lectin agarose beads as described [26]. Beads were washed 5 times in detergent buffer, and proteins were eluted with SDS-sample buffer for 20 min at 60°C. Samples were then subjected to 10% SDS-polyacrylamide gel electrophoresis and immunoblotted onto nitrocellulose. Blots were incubated with anti-CCR7 {1188, 1 µg/ml}, anti-CXCR4 {UMB-2, 1∶100}, anti-CXCR4 {6190, 1–10 µg/ml}, anti-CXCR4 {44716, 5–15 µg/ml} or anti-CXCR4 {12G5, 1–25 µg/ml} antibodies followed by the appropriate peroxidase-conjugated secondary antibodies and enhanced chemiluminescence detection (Amersham, Braunschweig, Germany). For all animal procedures, ethical approval was sought before the experiments according to national and institutional requirements.

### Immunohistochemistry

Seven µm paraffin sections were cut and floated onto positively charged slides and immunohistochemically stained as described. For antigen retrieval, sections were microwaved in 10 mM citric acid (pH 6.0) for 20 min at 600 W. Specimens were then incubated with anti-CXCR4 {UMB-2, 1∶10}, anti-CXCR4 {6190, 1–10 µg/ml}, anti-CXCR4 {44716, 5–15 µg/ml} or anti-CXCR4 {12G5, 1–25 µg/ml} antibodies overnight at 4°C. Staining of primary antibody was detected using the appropriate biotinylated secondary antibodies followed by incubation with avidin-biotinylated peroxidase solution. Tissue was then rinsed and stained with diaminobenzidine for 15 min. Cell nuclei were lightly counterstained with hematoxylin. For immunohistochemical controls, the primary antibody was either omitted or adsorbed with homologous or heterologous peptides for 2 h at room temperature.

## Results

### Characterization of Rabbit Monoclonal anti-CXCR4 Antibody UMB-2

**Figure 1:**
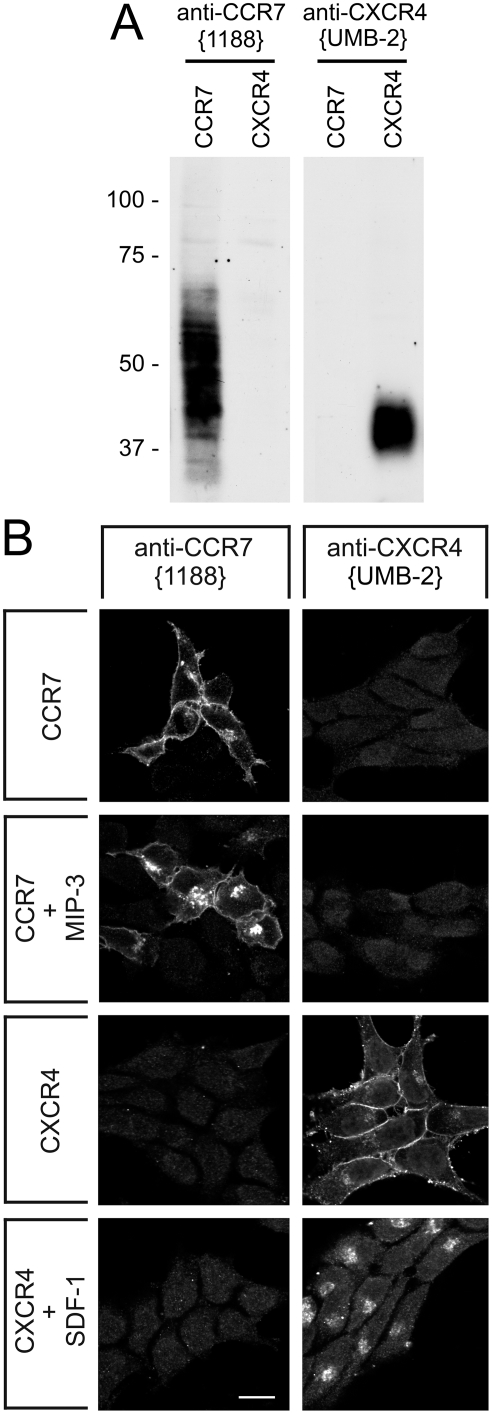
Characterization of rabbit monoclonal antibody UMB-2 using transfected cells. *A*, Western blot analysis of the specificity of anti-CXCR4 antibodies. Membrane preparations from HEK-293 cells stably transfected to express either CCR7 or CXCR4 were separated on 10% SDS-polyacrylamide gels and blotted onto nitrocellulose membranes. Membranes were then incubated with affinity-purified 1 µg/ml anti-CCR7 {1188} or anti-CXCR4 {UMB-2} hybridoma supernatant at a dilution of 1∶100. Blots were developed using enhanced chemiluminescence. Note that UMB-2 detected a band of the expected molecular weight only in CXCR4- but not in CCR7-transfected cells. Two additional experiments gave similar results. *Ordinate*, migration of protein molecular weight markers (*M*_r_×10^−3^). *B*, characterization of UMB-2 by immunofluorescent staining of transfected cells. HEK-293 cells expressing CCR7 or CXCR4 were either not exposed or exposed to 100 ng/ml MIP-3 or 100 ng/ml SDF-1 for 30 min, subsequently fixed and immunofluorescently stained with 1 µg/ml anti-CCR7 {1188} or anti-CXCR4 {UMB-2} at a dilution of 1∶100. Note that UMB-2 detected prominent immunofluorescence at the level of the plasma membrane only in CXCR4- but not in CCR7-expressing cells, and that SDF-1 exposure induced a rapid translocation of CXCR4 receptor immunostaining from the plasma membrane into the cytosol. Representative results from one of three independent experiments are shown. Scale bar, 20 µm.

Specificity of anti-CXCR4 antibody {UMB-2} was monitored using Western blot analysis of extracts prepared from CXCR4-expressing and CCR7-expressing HEK 293 cells. When membrane preparations from these cells were electrophoretically separated and blotted onto nitrocellulose, UMB-2 revealed a single broad band migrating at *M*_r_ 38,000 to 43,000 only in cells transfected with CXCR4 but not in CCR7-transfected cells (Figure 1 A, *right panel*). The rabbit polyclonal anti-CXCR4 antibodies {2144} and {1181} yielded identical results [26]. Conversely, the anti-CCR7 antibody {1188} detected a broad band migrating at *M*_r_ 40,000 to 60,000 only in CCR7-transfected cells but not in HEK-293 cells stably expressing CXCR4 (Figure 1 A, *left panel*). In contrast, the mouse monoclonal anti-CXCR4 antibody {12G5} and two other commercially available anti-CXCR4 antibodies were not able to detect the CXCR4 receptor in the same samples under identical conditions (Supplemental Figure S1). The rabbit monoclonal anti-CXCR4 antibody {UMB-2} was further characterized using immunofluorescent staining of transfected cells. When HEK-293 cells stably expressing CXCR4 or CCR7 were stained with UMB-2, prominent immunofluorescence localized at the level of the plasma membrane was selectively detected in cells expressing CXCR4 (Figure 1 B, *right panel*). After incubation with SDF-1, CXCR4 immunostaining was translocated from the plasma membrane into the cytosol indicating rapid agonist-induced endocytosis of the receptor (Figure 1 B, *right panel*). The rabbit polyclonal anti-CXCR4 antibodies {2144} and {1181} yielded identical results [26]. Conversely, the anti-CCR7 antibody {1188} detected its cognate receptor at the plasma membrane of CCR7-transfected cells but not in CXCR4-expressing cells (Figure 1 B, *left panel*). CCR7 immunostaining was translocated from the plasma membrane into the cytosol after exposure to MIP-3 (Figure 1 B, *left panel*). In contrast, under identical conditions none of the commercially available antibodies was able to discriminate between CXCR4- and CCR7 expressing cells (Supplemental Figure S1). Immunofluorescence was either not detectable {6190}, present at the cell nucleus {12G5} or at the cell surface {44716} but in each case completely unrelated to CXCR4 (Supplemental Figure S1).

**Figure 2:**
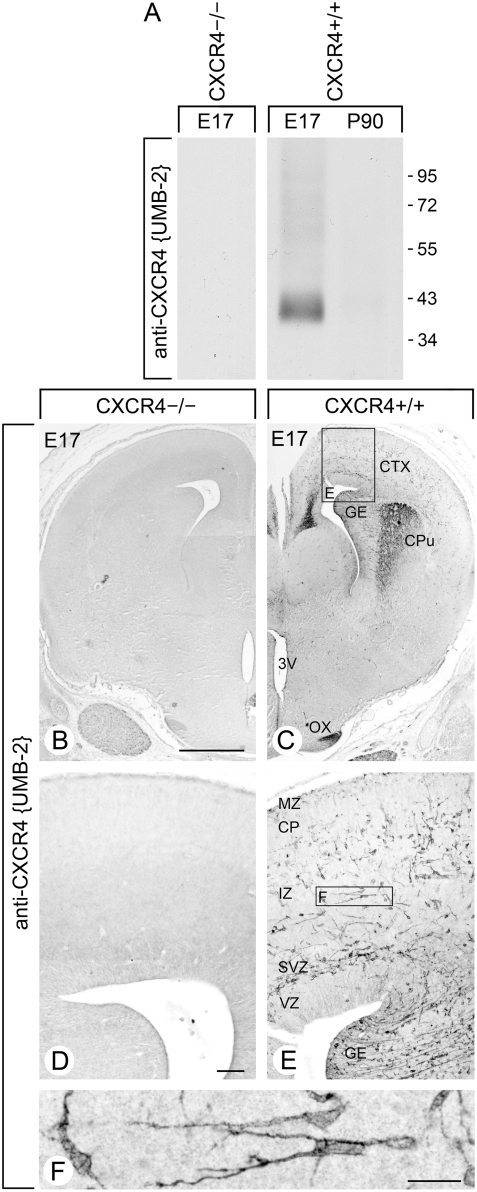
Characterization of rabbit monoclonal antibody UMB-2 using CXCR4-deficient mice. *A*, Western blot analysis of the specificity of UMB-2 using brain homogenates from CXCR4-deficient mice (CXCR4−/−) prepared at embryonic day 17 (E17) and their wild-type littermates (CXCR4+/+) as well adult CXCR4+/+ brain prepared at postnatal day 90 (P90). Note that UMB-2 selectively detects CXCR4 receptors at E17 in CXCR4+/+ mice and does not cross-react with other proteins present in tissue extracts prepared from CXCR4−/− mice. Migration of protein molecular weight markers (*M*_r_×10^−3^) is indicated on the right. *B–E*, characterization of UMB-2 by immunohistochemical staining of formalin-fixed, paraffin-embedded brain sections prepared at E17 from CXCR4-deficient mice (CXCR4−/−, *left panel*) and their wild-type littermates (CXCR4+/+, *right panel*). Note that UMB-2 selectively detects CXCR4 receptors and does not cross-react with other proteins present in tissue sections from CXCR4−/− mice. *F*, prominent CXCR4 receptor immunostaining at the plasma membrane of tangentially migrating neurons in the cortex. CTX, cortex; GE, ganglionic eminence; CPu, caudate putamen; OX, optic chiasm; 3V, third ventricle; MZ, marginal zone; CP, cortical plate; IZ, intermediate zone; SVZ, subventricular zone; VZ, ventricular zone. Scale bars, B = C = 500 µm, D = E = 100 µm, F = 50 µm.

Specificity of UMB-2 was then monitored in Western blot experiments using tissues from CXCR4-deficient mice and their wild-type littermates. The sequence used to generate UMB-2 is identical in mice, rats and humans. Using the rabbit polyclonal anti-CXCR4 antibodies {2144} and {1181}, we have previously shown that the CXCR4 receptor is expressed in distinct populations of neurons in the embryonic brain and that CXCR4 expression strongly declines during development [24], [25], [27]. In crude extracts from embryonic brain tissue (E17) of wild-type mice (CXCR4+/+), UMB-2 detected a broad band migrating at *M*_r_ 38,000 to 43,000, which corresponds to the expected size of the CXCR4 receptor (Figure 2 A, *right panel*). In contrast, UMB-2 did not detect any immunoreactive band in embryonic brain extracts from CXCR4-deficient mice (Figure 2 A, *left panel*) and only a very weak band in brain extracts from adult CXCR4+/+ mice (Figure 2 A, *right panel*). When forebrain sections of embryonic mice (E17) were immunohistochemically stained, UMB-2 yielded the well-known CXCR4 staining pattern with prominent immunoreactivity in the cortex, caudate putamen, ganglionic eminence in CXCR4+/+ mice but not in CXCR4−/− mice (Figure 2 B–E). At higher magnification it became apparent that CXCR4 immunostaining decorated the plasma membrane of tangentially migrating neurons in the cortex (Figure 2 F).

### UMB-2 Immunohistochemistry in Normal and Neoplastic Human Tissues

**Figure 3:**
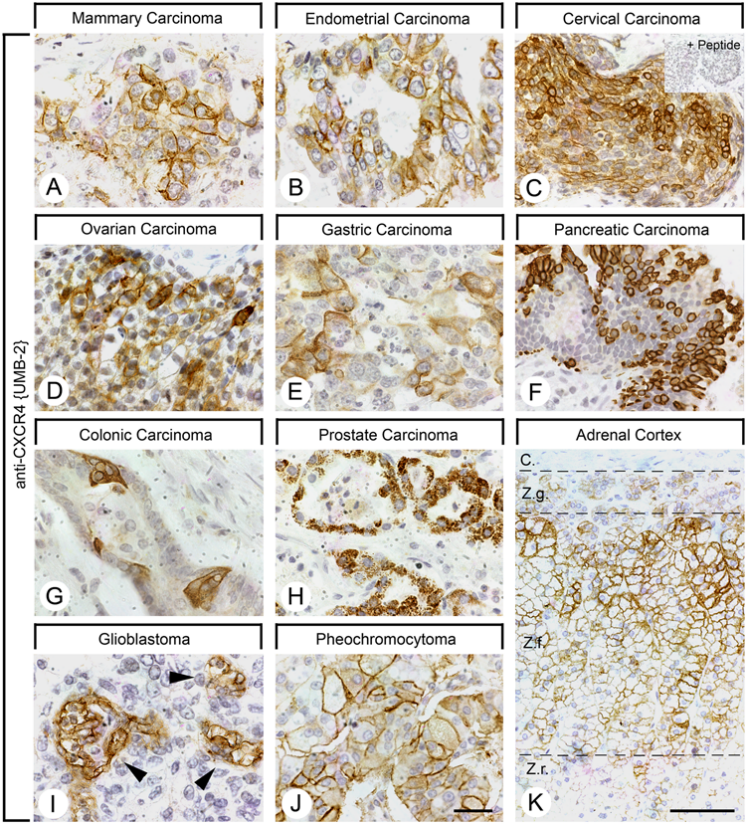
UMB-2 immunohistochemical staining in human formalin-fixed and paraffin-embedded tissues. *A*–*K*, UMB-2 immunohistochemical staining of a variety of human normal and neoplastic tissues. Sections were dewaxed, microwaved in citric acid and incubated with UMB-2 at a dilution of 1∶10. Sections were sequentially treated with biotinylated anti-rabbit IgG and AB solution. Sections were then developed in diaminobenzidine and lightly counterstained with hematoxylin. Note that UMB-2 detected CXCR4 receptors at the plasma membrane of a subset of tumor cells in a variety of human tumors including mammary carcinoma (A), endometrial carcinoma (B), cervical carcinoma (C), ovarian carcinoma (D), gastric carcinoma (E), pancreatic carcinoma (F), colonic carcinoma (G) and malignant pheochromocytoma (J). In nearly all of these cases UMB-2 immunoreactive tumor cells exhibited a heterogeneous distribution throughout the tumor. UMB-2 revealed predominant cytoplasmic staining in prostate carcinoma (H). In glioblastoma, UMB-2 detected CXCR4 receptors predominantly on the plasma membrane of endothelial cell of tumoral blood vessels (I). UMB-2 also detected CXCR4 receptors in the *zona fasciculata* of the adrenal cortex (K). Inset in C, peptide adsorption control. Arrowheads in I, tumoral blood vessels. C, capsule; Z.g., Zona glomerulosa; Z.f., Zona fasciculata; Z.r., Zona reticularis. Scale bars, A = B = C = D = E = F = G = H = I = J = K = 50 µm, K = 100 µm.

First, UMB-2 was tested on a variety of formalin-fixed, paraffin-embedded human breast cancers, which have previously been reported to express CXCR4 [2]. Initial experiments revealed that UMB-2 yielded highly effective immunohistochemical staining of fixed-embedded tissues with a predominance of plasma membrane staining and very low cytoplasmic signal (Figure 3 A). Initial experiments also demonstrated that UMB-2 detected CXCR4 receptors in fixed-embedded human tissues even without heat-based antigen retrieval (not shown). In most cases UMB-2 immunostaining was not uniformly present on all tumor cells but rather heterogeneously distributed throughout the tumor (Figure 3). UMB-2 immunostaining was completely abolished by preadsorption of the antibody with 10 µg/ml of its immunizing peptide (Figure 3 C).

A series of 247 human tissue samples was then immunohistochemically stained with UMB-2. Prominent CXCR4 staining of the tumor cells was observed in a number of mammary, pancreatic, prostate, cervical and ovarian carcinomas (Table 1, Figure 3). UMB-2 immunostaining was not detected in GH-secreting pituitary adenomas and pheochromocytomas except for one case of a malignant pheochromocytoma (Table 1, Figure 3). As depicted in Figures 3 A, B, C, D, F, G, and J robust plasma membrane staining of tumor cells was most frequently observed. In contrast, a number of prostate carcinomas presented with punctuate cytoplasmic staining (Figure 3 H). However, in virtually none of our cases CXCR4 receptors were detected at cell nuclei (Figure 3). CXCR4 was also frequently detected on tumoral micro vessels (Table 1). In our cases, CXCR4 was not observed on tumor cells in astrocytomas or glioblastomas. It should be noted, however, that CXCR4 was found on a high density on vascular endothelial cells in glioblastoma multiforme but never in astrocytomas grade I or II. UMB-2 also facilitated detection of CXCR4 receptors in non-neoplastic tissues, i.e. prominent UMB-2 immunostaining was seen in the *zona fasciculata* of the adrenal cortex but not in the adrenal medulla (Figure 3 K).

**Table 1:** Prevalence of CXCR4 receptors in human tumors.

Tumor type (n)	CXCR4 n (%)	Tumoral blood vessels CXCR4 pos. n (%)
Mammary carcinoma (36)	13 (36)	4 (11)
Ovarian carcinoma (22)	8 (36)	0
Cervical carcinoma (16)	6 (38)	0
Endometrial carcinoma (4)	1 (25)	0
Gastric carcinoma (13)	4 (30)	2 (15)
Colorectal carcinoma (23)	5 (22)	1 (4)
Pancreatic carcinoma (29)	16 (55)	11 (38)
Prostate carcinoma (24)	19 (79)	0
Carcinoid (18)	7 (39)	1 (6)
GH-adenoma (8)	0	0
Pheochromocytoma (20)	1 (5)	2 (10)
Astrocytoma grade II (8)	0	0
Astrocytoma grade III (8)	0	0
Glioblastoma multiforme (18)	0	16 (89)

Finally, the rabbit monoclonal anti-CXCR4 antibody {UMB-2} was compared with mouse monoclonal antibody 12G5 and other frequently used commercially available antibodies. As depicted in Supplemental Figure S2, UMB-2 immunohistochemistry allowed a clear discrimination between CXCR4-positive and CXCR4-negative tumors. In contrast, under identical conditions commercially available antibodies yielded either no detectable immunostaining {6190} or prominent staining of cell nuclei {44716} and/or occasional cytoplasmic staining {12G5} (Supplemental Figure S2).

## Discussion

In an effort to study the pattern of CXCR4 receptor protein expression in human normal and neoplastic tissues, we extensively characterized the novel rabbit monoclonal anti-CXCR4 antibody {UMB-2}. Our results demonstrate that the cytoplasmic tail of the CXCR4 receptor can serve as an epitope for the generation of monoclonal antibodies that effectively stain formalin-fixed, paraffin-embedded human and rodent tissues. Several lines of evidence indicate that UMB-2 specifically detects its targeted receptor and does not crossreact. First, in Western blots of membranes from CXCR4-transfected cells UMB-2 detected a broad band migrating at *M*_r_ 38,000–43,000. Second, UMB-2 revealed prominent cell surface staining of CXCR4-transfected cells. This immunostaining translocated from the cell surface into the cytosol after SDF-1 exposure. Third, UMB-2 clearly detected CXCR4 receptors in homogenates and tissue sections from wild-type mice but not in tissues from CXCR4-deficient mice. Fourth, UMB-2 detected bona fide plasma membrane receptors in fixed-embedded human tissues. Finally, UMB-2 immunostaining was completely abolished by preadsorption with its immunizing peptide.

In contrast to currently available anti-CXCR4 antibodies, the novel rabbit monoclonal antibody UMB-2 yielded highly effective plasma membrane staining of tumor cells without any detectable immunostaining of cell nuclei. The plasma membrane localization would be compatible with our understanding of CXCR4 function in cancer cell migration and homing [1]. However, it should be noted that in the majority of tumors CXCR4 immunostaining was heterogeneously distributed throughout the tumor and not uniformly present on all tumor cells. Interestingly, CXCR4 immunostaining was also frequently observed in tumoral micro vessels in glioblastoma multiforme but not in astrocytomas. The use of UMB-2 also permitted us to gain novel insights into CXCR4 receptor expression in normal tissues, e.g. CXCR4 was detected for the first time in the Zona fasciculata of the adrenal cortex. However, the precise function of CXCR4 in the regulation cortisol production or secretion needs to be established.

The rabbit monoclonal antibody UMB-2 will overcome a number of limitations of currently available antibodies, e.g. the most frequently used commercially available anti-CXCR4 antibodies have only been selected for their binding to native receptors on living cells and have not been adequately characterized on fixed cells and tissues [21], [22]. In addition, only limited epitope information is available which prevents the inclusion of adsorption controls during immunohistochemical staining [21], [22]. Thus, the development of UMB-2 will now allow the establishment of guidelines for the routine performance and interpretation of CXCR4 immunohistochemistry in a variety of human tumors.

## Supporting Information

Figure S1 Comparative analysis of UMB-2 and currently available CXCR4 antibodies using transfected cells. A, Western blot analysis of the specificity of anti-CXCR4 antibodies. Membrane preparations from HEK-293 cells stably transfected to express either CCR7 or CXCR4 were separated on 10% SDS-polyacrylamide gels and blotted onto nitrocellulose membranes. Membranes were then incubated with 1 µg/ml anti-CCR7 {1188}, anti-CXCR4 {UMB-2} hybridoma supernatant at a dilution of 1∶100, 10 µg/ml anti-CXCR4 {6190}, 15 µg/ml anti-CXCR4 {44716} or 25 µg/ml anti-CXCR4 {12G5} antibodies. Blots were developed using enhanced chemiluminescence. Note that only UMB-2 detected the appropriate CXCR4 receptor band. Ordinate, migration of protein molecular weight markers (Mr×10−3). B, characterization of CXCR4 antibodies by immunofluorescent staining of transfected cells. B, HEK-293 cells stably transfected to express CCR7 or CXCR4 were either not exposed or exposed to 100 ng/ml MIP-3 or 100 ng/ml SDF-1 for 30 min, and subsequently fixed and immunofluorescently stained with 1 µg/ml anti-CCR7 {1188}, anti-CXCR4 {UMB-2} at a dilution of 1∶100, 10 µg/ml anti-CXCR4 {6190}, 15 µg/ml anti-CXCR4 {44716} or 25 µg/ml anti-CXCR4 {12G5} antibodies. UMB-2 selectively detected plasma membrane immunofluorescence in CXCR4-expressing cells that rapidly translocated into the cytosol after SDF-1 exposure. Note that none of the commercially available antibodies was able to detect CXCR4 in fixed cells or cell lysates under otherwise identical conditions. Representative results from one of two independent experiments are shown. Scale bar, 20 µm.
(3.80 MB TIF)

Figure S2 Comparative analysis of immunohistochemical staining of human tissues using UMB-2 and currently available CXCR4 antibodies. Adjacent sections of a variety of human tumors were dewaxed, microwaved in citric acid and incubated with anti-CXCR4 {UMB-2} at a dilution of 1∶10, 10 µg/ml anti-CXCR4 {6190}, 15 µg/ml anti-CXCR4 {44716} or 25 µg/ml anti-CXCR4 {12G5} antibodies. Sections were sequentially treated with biotinylated secondary antibodies AB solution. Sections were then developed in diaminobenzidine and lightly counterstained with hematoxylin. Note that UMB-2 immunohistochemistry allowed a clear distinction between CXCR4 positive and CXCR4 negative tumors. Whereas anti-CXCR4 {44716} and anti-CXCR4 {12G5} produced a predominant nuclear staining, anti-CXCR4 {6190} produced no staining under otherwise identical conditions. Scale bar 50 µm.
(7.57 MB TIF)
