# Supplementary figures and images for: Reassessment of CXCR4 Chemokine Receptor Expression in Human Normal and Neoplastic Tissues Using the Novel Rabbit Monoclonal Antibody UMB-2
Source: PLoS One. 2008 Dec 31;3(12):e4069. doi: 10.1371/journal.pone.0004069 (PMC2605258; doi:10.1371/journal.pone.0004069)

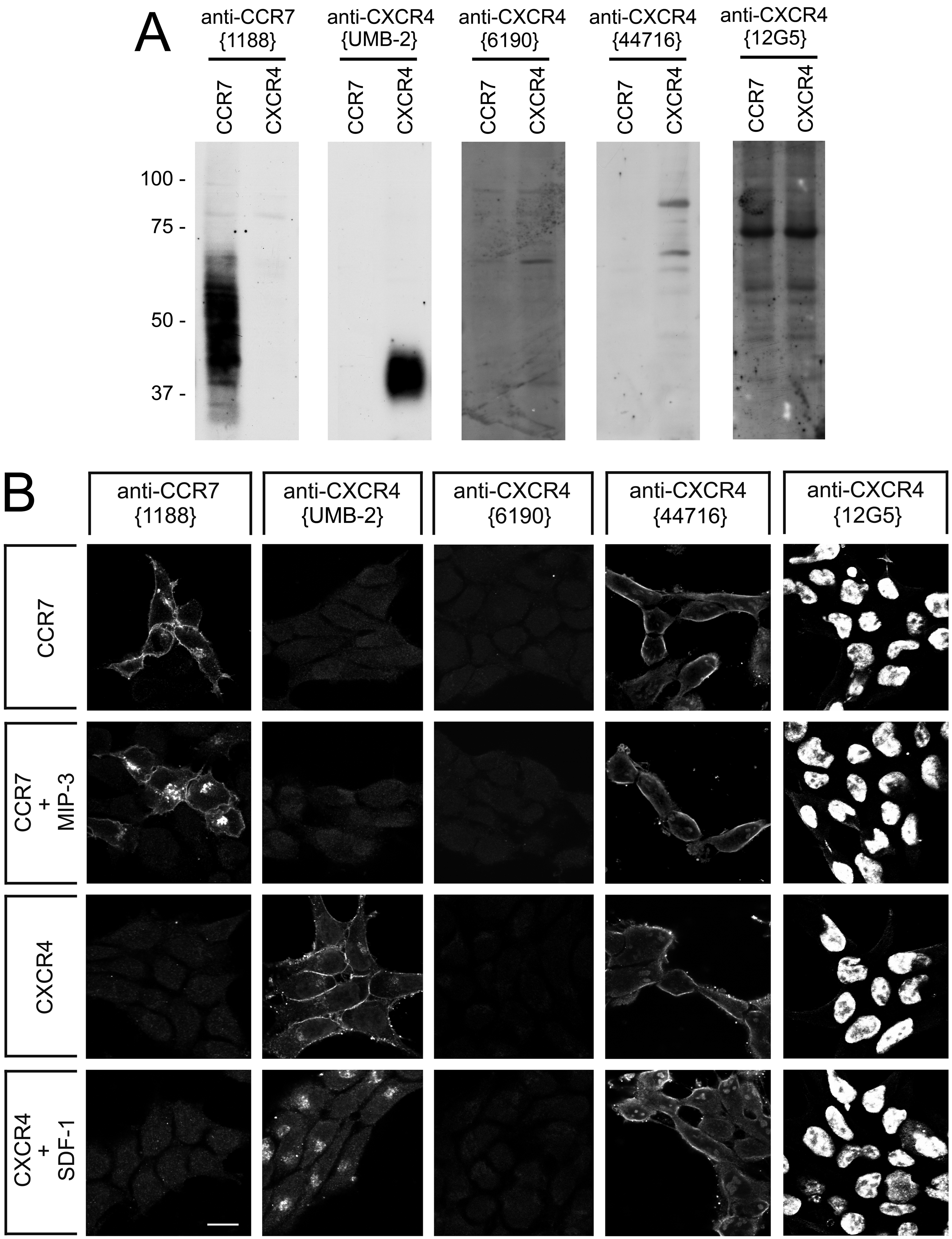

Supplement: Figure S1 — Comparative analysis of UMB-2 and currently available CXCR4 antibodies using transfected cells. A, Western blot analysis of the specificity of anti-CXCR4 antibodies. Membrane preparations from HEK-293 cells stably transfected to express either CCR7 or CXCR4 were separated on 10% SDS-polyacrylamide gels and blotted onto nitrocellulose membranes. Membranes were then incubated with 1 µg/ml anti-CCR7 {1188}, anti-CXCR4 {UMB-2} hybridoma supernatant at a dilution of 1∶100, 10 µg/ml anti-CXCR4 {6190}, 15 µg/ml anti-CXCR4 {44716} or 25 µg/ml anti-CXCR4 {12G5} antibodies. Blots were developed using enhanced chemiluminescence. Note that only UMB-2 detected the appropriate CXCR4 receptor band. Ordinate, migration of protein molecular weight markers (Mr×10−3). B, characterization of CXCR4 antibodies by immunofluorescent staining of transfected cells. B, HEK-293 cells stably transfected to express CCR7 or CXCR4 were either not exposed or exposed to 100 ng/ml MIP-3 or 100 ng/ml SDF-1 for 30 min, and subsequently fixed and immunofluorescently stained with 1 µg/ml anti-CCR7 {1188}, anti-CXCR4 {UMB-2} at a dilution of 1∶100, 10 µg/ml anti-CXCR4 {6190}, 15 µg/ml anti-CXCR4 {44716} or 25 µg/ml anti-CXCR4 {12G5} antibodies. UMB-2 selectively detected plasma membrane immunofluorescence in CXCR4-expressing cells that rapidly translocated into the cytosol after SDF-1 exposure. Note that none of the commercially available antibodies was able to detect CXCR4 in fixed cells or cell lysates under otherwise identical conditions. Representative results from one of two independent experiments are shown. Scale bar, 20 µm. (3.80 MB TIF) [file pone.0004069.s001.tif]

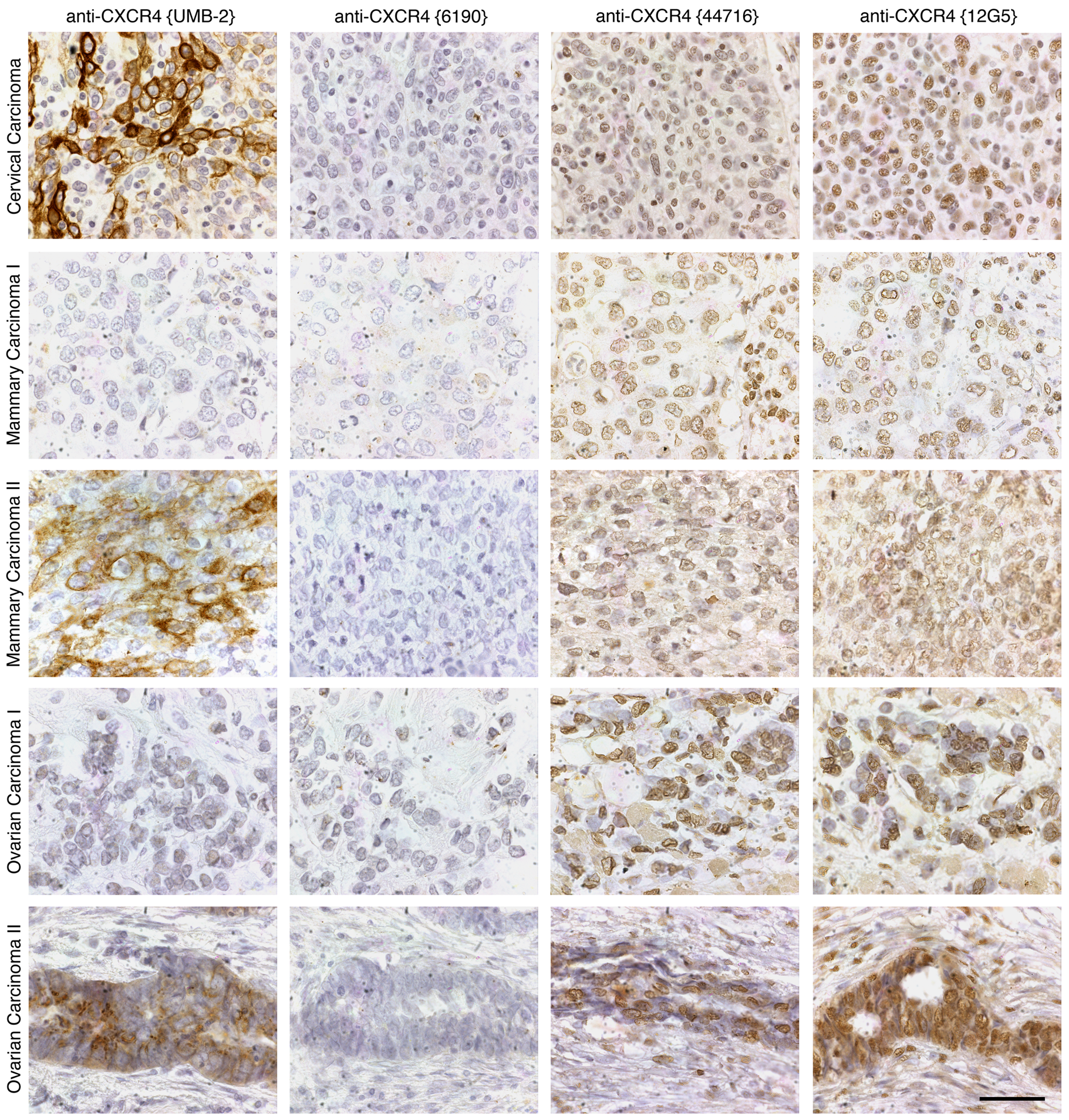

Supplement: Figure S2 — Comparative analysis of immunohistochemical staining of human tissues using UMB-2 and currently available CXCR4 antibodies. Adjacent sections of a variety of human tumors were dewaxed, microwaved in citric acid and incubated with anti-CXCR4 {UMB-2} at a dilution of 1∶10, 10 µg/ml anti-CXCR4 {6190}, 15 µg/ml anti-CXCR4 {44716} or 25 µg/ml anti-CXCR4 {12G5} antibodies. Sections were sequentially treated with biotinylated secondary antibodies AB solution. Sections were then developed in diaminobenzidine and lightly counterstained with hematoxylin. Note that UMB-2 immunohistochemistry allowed a clear distinction between CXCR4 positive and CXCR4 negative tumors. Whereas anti-CXCR4 {44716} and anti-CXCR4 {12G5} produced a predominant nuclear staining, anti-CXCR4 {6190} produced no staining under otherwise identical conditions. Scale bar 50 µm. (7.57 MB TIF) [file pone.0004069.s002.tif]
